# CEACAM1 Activation by CbpF-Expressing *E. coli*

**DOI:** 10.3389/fcimb.2021.699015

**Published:** 2021-07-29

**Authors:** Amjad Shhadeh, Johanna Galaski, Tamar Alon-Maimon, Jamal Fahoum, Reuven Wiener, Daniel J. Slade, Ofer Mandelboim, Gilad Bachrach

**Affiliations:** ^1^The Institute of Dental Sciences, The Hebrew University-Hadassah School of Dental Medicine, Jerusalem, Israel; ^2^The Concern Foundation Laboratories at the Lautenberg Center for General and Tumor Immunology, Department of Immunology and Cancer Research, Institute for Medical Research Israel Canada (IMRIC), Faculty of Medicine, The Hebrew University Medical School, Jerusalem, Israel; ^3^I. Department of Medicine, University Medical Center Hamburg-Eppendorf, Hamburg, Germany; ^4^Department of Biochemistry and Molecular Biology, Institute for Medical Research Israel Canada (IMRIC), Faculty of Medicine, The Hebrew University Medical School, Jerusalem, Israel; ^5^Department of Biochemistry, Virginia Polytechnic Institute and State University, Blacksburg, VA, United States

**Keywords:** *F. nucleatum*, CbpF, trimeric autotransporter adhesin, CEACAM1, NK cells

## Abstract

Recent studies on the oral, anaerobic, gram-negative bacterium *Fusobacterium nucleatum* revealed its presence and involvement in colorectal, esophageal and breast cancer. We previously demonstrated that *F. nucleatum* binds and activates the human inhibitory receptors TIGIT and CEACAM1 leading to inhibition of T and NK cell anti-tumor immunity. CEACAM1 was found to be bound and activated by the fusobacterial trimeric autotransporter adhesin CbpF. Here we report the generation of a recombinant *E. coli* expressing full-length CbpF that efficiently binds and activates CEACAM1.

## Introduction

*Fusobacterium nucleatum* is an oral, gram-negative, anaerobic bacterium and one of the most abundant species found in the oral cavity (Socransky et al., 1998; Nozawa et al., 2020). It is one of the phathobionts (Hajishengallis and Lamont, 2016; Lamont et al., 2018) that outgrow during dysbiosis preceding periodontal disease (Socransky et al., 1998; Nozawa et al., 2020). *F. nucleatum* is also found in colon adenocarcinoma (Castellarin et al., 2012; Kostic et al., 2012), esophageal cancer (Yamamura et al., 2016), pancreatic cancer (Mitsuhashi et al., 2015) and breast cancer (Parhi et al., 2020). The presence of *F. nucleatum* in pancreatic colon and esophageal cancer has been associated with poor prognosis (Mitsuhashi et al., 2015; Mima et al., 2016; Yamamura et al., 2016; Yamaoka et al., 2018). Generation of a pro-tumorigenic immune microenvironment (Kostic et al., 2013) and inhibition of the recruitment of tumor infiltrating lymphocytes (TILs) (Mima et al., 2015; Parhi et al., 2020) are among the mechanisms manipulated by *F. nucleatum* to promote cancer development. In addition to inhibition of the recruitment of TILs to the tumor, *F. nucleatum* activates the T and natural killer (NK) cells inhibitory receptors TIGIT and CEACAM1, leading to a reduction in their ability to kill tumor cells (Gur et al., 2015; Brewer et al., 2019; Gur et al., 2019b; Galaski et al., 2021).

CEACAM1 is a member of the human carcinoembryonic antigen-related cell adhesion molecules (CEACAMs) that mediate cell-cell interactions and cellular signaling events. On various immune cell subsets, CEACAM1 acts as an inhibitory receptor (Gray-Owen and Blumberg, 2006; Yamin et al., 2016; Gur et al., 2019a; Gur et al., 2019b). The checkpoint-inhibitory activity of CEACAM1 can be activated by several ligands: CEACAM1 (CEACAM1-CEACAM1 interactions); Opacity-associated (Opa) proteins of pathogenic Neisseria (Gray-Owen and Blumberg, 2006); ubiquitous surface proteins A1 (UspA1) and A2V (UspA2V) of *Moraxella catarrhalis* (Hill and Virji, 2003; Hill et al., 2012); HopQ of *Helicobacter pylori* (Javaheri et al., 2016; Koniger et al., 2016; Gur et al., 2019a; Tegtmeyer et al., 2019) and by the CEACAM binding protein of *Fusobacterium* (CbpF) that facilitates fusobacterial colonization in the CEACAM1 displaying dentogingival junction (Brewer et al., 2019), and activates CEACAM1 signaling in immune cells (Galaski et al., 2021).

CEACAM1 is expressed on cells of the myeloid lineage, epithelial cells of human mucosa and some endothelial cells (Prall et al., 1996; Obrink, 1997; Hammarstrom, 1999). In addition, it is found on a wide variety of tumor cells, where it is considered to be a biomarker correlated with tumor progression, metastasis and poor prognosis (Wang et al., 2014; Dankner et al., 2017).

Tumor infiltrating lymphocytes (TILs) found in the colorectal cancer microenvironment are characterized by high levels of CEACAM1, along with reduced levels of cytotoxic activity when compared to paraneoplastic T cells (Zhang et al., 2017). These findings suggest a role of CEACAM1 in mediating T cell exhaustion. The homophilic interactions of CEACAM1, occurring between CEACAM1^+^ TILs, and CEACAM1^+^ tumor cells, as well as the interaction of CbpF with CEACAM1 expressed on NK and T cells, may protect tumors from killing by these immune cells in a synergistic mechanism.

Analysis of the CbpF amino acid sequence indicated that it belongs to the autotransporter proteins superfamily of the type Vc secretion pathway (Brewer et al., 2019). All autotransporters contain a three-domain structure which include an N-terminal leader peptide, a passenger domain and a C-terminal β-barrel domain. The C-terminal β-barrel autotransporter domain creates a β-barrel channel in the bacterial outer membrane that allows the passage of the passenger domain through the outer membrane to be presented on the bacterial outer surface (Henderson et al., 2004). Unlike other autotransporter subfamilies, the members of the type Vc secretion systems, often referred to as trimeric autotransporter adhesins (TAAs), form trimeric autotransporter proteins that consist of three identical polypeptide chains (Leo et al., 2012).

The C-terminus hydrophobic β-barrel domain of autotransporters can lead to solubility issues during the production of full-length recombinant autotransporter proteins. To the best of our knowledge, TAAs have most often been studied using truncated recombinant proteins expressed in *E. coli* (Nordstrom et al., 2004; Riess et al., 2004; Tan et al., 2005; Leo et al., 2008; Szczesny et al., 2008; Leo and Goldman, 2009; Hallstrom et al., 2011; Leo et al., 2011; Bentancor et al., 2012; Brewer et al., 2019). The features of truncated recombinant proteins might not fully match those of the native full-length proteins. Therefore, studies on a full-length recombinant TAA might expand our knowledge about the function and molecular properties of these protein family members.

## Materials and Methods

### Bacterial Strains and Cell Lines Growth Conditions

*Escherichia coli* strains were grown in LB broth (Difco) or on LB agar plates (Difco), at 37°C under aerobic conditions.

*E. coli* strain C43(DE3) was used to express the CbpF variants, and strain Top10 was used to amplify the CbpF variants plasmids. All plasmids-transformed *E. coli* strains were grown in the presence of 100 µg/ml ampicillin (Sigma).

721.221 cells and BW cells were grown in RPMI supplemented with 10% heat-inactivated fetal calf serum (FCS), 1% each of non-essential amino acids, L-glutamine, sodium pyruvate and with penicillin-streptomycin (all from Biological Industries). Cells were grown at 37°C in a humidified 5% CO_2_ incubator.

### Plasmid Transformation and Purification

100 ng of recombinant plasmid was gently mixed with 100 μl chemically competent bacteria. After 30 min incubation on ice, the mixture was heat shocked at 42°C for 60 seconds and then placed on ice for 2 min. Next, 900 μl of LB medium was added to the cell mixture and tubes incubated for 1 h at 37°C with shaking at 220 rpm. Next, 100 μl of the transformation mixture were spread on LB agar plates supplemented with 100 μg/ml ampicillin.

For plasmid purification, single colonies of transformed-*E. coli* Top10 were inoculated into 5ml selective LB medium and cultivated overnight at 37°C with shaking at 220 rpm. Plasmid was extracted using the QIAprep Spin Miniprep Kit (Qiagen), according to the manufacturer’s instructions.

### Protein Expression and Purification

For expression, *E. coli* C43(DE3) was transformed with either the rCbpF-1 or rCbpF-2 encoding plasmids pSA-OCbpF1 and pSA-OCbpF2 respectively. A single colony was then inoculated in LB broth and grown overnight. The starter culture was then diluted x 100 in 2xYT medium (16 g/L tryptone, 10 g/L yeast extract, 5 g/L NaCl) containing, 100 µg/ml ampicillin (Sigma), 1 x NPS buffer (3.3 g/L (NH_4_)_2_SO_4_, 6.8 g/L KH_2_PO_4_, 7.1 g/L Na_2_HPO_4_) and 0.1% glucose, and grown at 37 °C until reaching an OD 600nm of 0.6–0.8. Cultures were then induced with 0.4 mM isopropyl-β-d-thio-galactoside (IPTG, Sigma) at 22°C overnight while shaking at 220 rpm. The cells were harvested by centrifuging at 5000 x g for 15 min, growth medium and part of the cell pellets were stored at -20°C until further use. The retained pellets were then resuspended with 1ml Lysis Buffer (50mM Tris pH 8.0, 10% glycerol, 1% Triton X-1 and 100 µg/ml lysozyme) and incubated 30 minutes in room temperature. Samples were then rapidly frozen by immersion in liquid nitrogen (2 min) and thawed in 42°C for 5 min. This freeze-thaw procedure was repeated three times, and the samples were centrifuged at 8,000 x g for 5 minutes at 4°C. The supernatant was transferred to a new test tube and both the pellet and the supernatant were kept also at -20°C for further analysis.

For rCbpF-1 purification, the bacterial growth medium was filtered with a 0.2 µm vacuum filter (Merck Millipore) and then concentrated x80 using a 50 kDa centrifugal filter (Amicon Ultra – 15, Merck Millipore). The concentrated growth medium was then loaded on to the 5 ml His-Trap columns (GE Healthcare), washed with washing buffer (50 mM NaH_2_PO_4_, 500 mM NaCl, 10 mM imidazole, pH 8.0), and eluted with a linear gradient of imidazole (15–300 mM). Fractions containing pure proteins were pooled and dialyzed against a buffer containing 20 mM Tris pH 7.5, 150 mM NaCl, 10% glycerol.

### Gel Electrophoresis

Samples were dissolved in a denaturing sample buffer (192 mM Tris-HCl [pH 6.8], 30% glycerol, 9% SDS, 0.01% bromophenol blue, 2% β-mercaptoethanol), or in a non-reducing sample buffer (lacking β-mercaptoethanol). The denaturing samples were also boiled at 95°C for 10 minutes, and samples were subjected to SDS-PAGE gels. Following electrophoresis, the gels were stained with Coomassie brilliant blue R-250 (Bio-Rad).

### Western Blot and Far Western

Following electrophoresis, the proteins were transferred to a nitrocellulose membrane (110 V, 1 h, 4°C) using the Mini Trans-Blot cell apparatus (Bio-Rad). Nonspecific binding sites were blocked using PBS-Tween 20 (0.05% Tween 20, J.T. Baker) containing 5% non-fat dry milk for 1 h at room temperature. The membrane was then overlaid with mouse anti-His antibody (Bio-Rad), diluted 1:1000 in the blocking solution and incubated overnight at 4°C. Next day, the membrane was washed three times with PBS-Tween 20 and overlaid with peroxidase-conjugated goat anti-mouse antibodies (Jackson ImmunoResearch Laboratories) diluted 1:5000 in blocking solution, for 1 h at room temperature. The membrane was then washed three times with PBS-Tween 20. Before imaging (using the ChemiDoc MP imaging system, Bio-Rad), the membrane was incubated in EZ-ECL (Biological Industries) solution for 5 min.

For far western blotting, the experiment was performed similar to the western blot experiment, but CEACAM1-Ig (2 µg/ml) (Gur et al., 2019b) was used instead of the first antibody and the HRP-conjugated α - human IgG (Jackson ImmunoResearch Laboratories, diluted 1:5,000) was used as a secondary antibody.

### FITC Labeling of Bacteria and Flow Cytometry

For FITC-labeling, bacteria were washed twice in PBS, and incubated with 0.1 mg/ml FITC (Sigma) in PBS at room temperature in the dark for 30 minutes. Subsequently, bacteria were washed three times in PBS to remove unbound FITC. For flow cytometry, 721.221 cells were used as carrier cells to facilitate gating. To this end, bacteria were divided into 96-well plates and incubated with 721.221 cells for 30 minutes on ice to allow for bacterial adhesion to the cells (6 x 10^7^ bacteria were placed together with 1 x 10^5^ 721.221 cells per well). Next, cells were washed and incubated with 3 µg of CEACAM1-Ig on ice for 1 hour followed by a 30-minute incubation with Alexa Fluor 647-conjugated donkey anti-human IgG (Jackson ImmunoResearch Laboratories). Histograms of cell-bound bacteria stained with CEACAM1-Ig were gated on FITC-positive cells.

### BW Assay

The generation of BW cells expressing chimeric CEACAM1 (composed of the extracellular portion of human CEACAM1 fused to the mouse CD3ζ chain) was previously described (Markel et al., 2002).

*E. coli* were inactivated at 60°C for 40 minutes, divided into 96-well plates (3x10^7^ bacteria per well) and incubated for one hour at 37°C in complete RPMI. Subsequently, BW CEACAM1 cells were added at 5x10^4^ per well and incubated with the bacteria for 48 hours at 37°C. Next, supernatants were collected and mouse IL-2 levels were quantified by a sandwich ELISA.

## Results

### Production of a Recombinant Trimeric CbpF Autotransporter Adhesin

In an attempt to obtain a functional recombinant CbpF, we synthesized genes for two new CbpF variants (rCbpF-1 and rCbpF-2). As cloning and expression of AT-rich DNA in *E. coli* is difficult (Coppenhagen-Glazer et al., 2015), and the fusobacterial genome possesses a high (~ 70%) AT content (Kapatral et al., 2002), both genes were optimized for *E. coli* by performing codon optimization (Genscript). In addition to codon optimization, in *cbpF*-1, the fusobacterial CbpF signal peptide was replaced with that of the *E. coli* OmpA. In *cbpF*-2 the last four amino acids of the fusobacterial CbpF signal peptide were kept, followed by the CbpF passenger domain. Thus the CbpF autotransporter β-barrel domain was omitted as described previously (Brewer et al., 2019). The optimized genes (shown in **Supplementary Figure 1**) were cloned into pET-11a to generate pSA-OCbpF1 and pSA-OCbpF2, respectively (**Supplementary Figure 2**).

**Figure 1:**
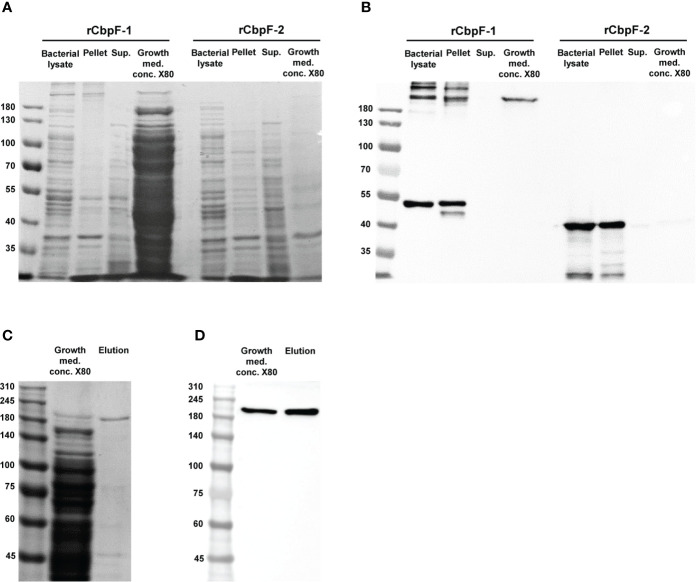
Expression of *cbpF*-1 and *cbpF*-2, and purification of rCbpF-1. *cbpF*-1 and *cbp*F-2 were expressed in *E. coli* C43 transformed with pSA-OCbpF1 and pSA-OCbpF2 respectively. Whole bacterial lysate, Pellet and Supernatant (Sup.) fractions after bacterial lysis, and growth medium concentrated X80 (Growth med. conc. X80) of *E. coli* C43 expressing either *cbpF*-1 or *cbpF*-2 were subjected to 7.5% gels SDS-PAGE **(A)**, and Western immunodetection using anti-His monoclonal antibody **(B)**. Recombinant CbpF-1 (rCbpF-1) was purified using Ni-NTA from growth medium concentrated X80 and subjected to 6% SDS-PAGE **(C)**, and Western immunodetection using anti-His monoclonal antibody **(D)**. Molecular weights are indicated on the left.

Expression of *cbpF*-1 in *E. coli* C43 resulted in the secretion of a full-length recombinant trimeric CbpF-1(rCbpF-1) to the growth medium, while *cbpF*-2 expression resulted in an intracellular monomeric truncated variant which required bacterial lysis for purification (**Figures 1A, B**). Next, rCbpF-1 was purified from *cbpF*-1 -expression growth medium concentrated 80 - fold (Growth med. conc. X80) using Ni-NTA (**Figures 1C, D**).

Similar to native CbpF expressed by *F. nucleatum* (Brewer et al., 2019), rCbpF-1 is a stable trimer that migrates in SDS-PAGE predominantly in a trimeric form following heating in reducing conditions.

### CbpF-1-Expressing Recombinant *E. coli* Binds and Activates CEACAM1

**Figure 2:**
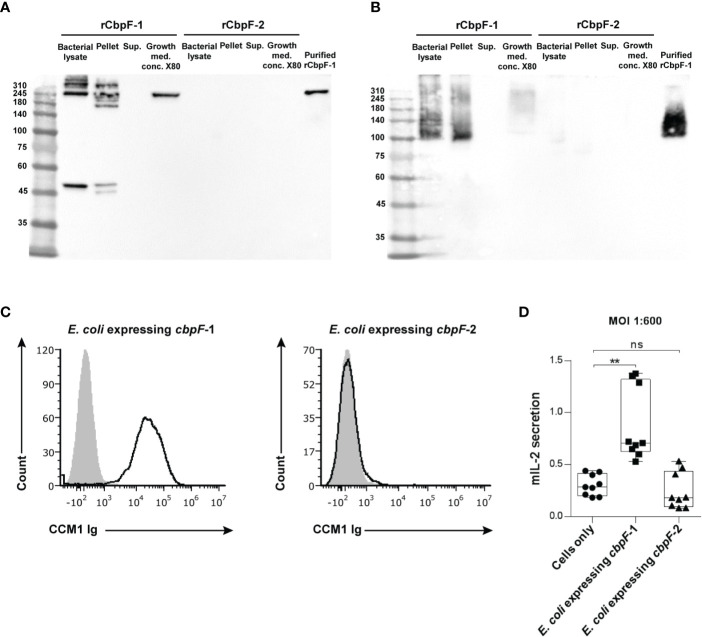
Recombinant *E. coli* expressing CbpF-1 binds CEACAM1. Whole bacterial lysate; Pellet and Supernatant (Sup.) fractions after lysis; growth medium concentrated X80 (Growth med. conc. X80) of *E. coli* C43 expressing *cbpF*-1 or *cbpF*-2; and Ni-NTA -purified rCbpF1 were subjected to denaturing **(A)**, and native **(B)** 7.5% SDS-PAGE followed by far western analysis using CEACAM1-Ig (see materials and methods). Molecular weights are indicated on the left. **(C)** FITC-labeled *E. coli* expressing *cbpF*-1 or *E. coli* expressing *cbpF*-2 were stained with 2 μg of human CEACAM1-Ig. Human B lymphoblastoid cell line 721.221 cells were used as carrier cells. Filled grey histograms represent staining with secondary antibody only. Presented results represents one of two independent repeats. **(D)** CEACAM1-reporter BW cells were incubated with *E. coli* expressing *cbp*F-1 or *cbp*F-2 at a ratio of 1:600. Mouse IL-2 (mIL-2) in the supernatants 48 hours later was determined by ELISA. Boxplot whiskers represent extrema, box bounds represent upper and lower quartiles, and center-line represents the median value of nine observations. n.s., non-significant, **p = 0.0033 two-tail, as determined by the Dunn *post-hoc* multiple-comparisons procedure following the Kruskal-Wallis test (GraphPad Prism 6).

Far western analysis under denaturing (**Figure 2A**) and non-reducing (**Figure 2B**) conditions was performed in order to test if the recombinant CbpF variants can bind CEACAM1. A CEACAM1-Ig fusion protein in which the extracellular domain of human CEACAM1 is fused to the Fc portion of human IgG1 (CEACAM1-Ig) was used as a probe. While the rCbpF-2 monomer failed to bind CEACAM1-Ig, the *E. coli*-bound CbpF-1 trimer, and the Ni-NTA -purified, secreted rCbpF-1 trimer were able to bind CEACAM1-Ig in both denaturing and native far western assays.

Flow cytometry using CEACAM1-Ig was used next to confirm that rCpbF-1 is displayed on the surface of *cbpF*-1 -expressing *E. coli*. As can be seen in **Figure 2C**, binding of human CEACAM1-Ig was observed only to *cbpF*-1-expressing *E. coli* but not to *cbpF*-2-expressing ones.

We next wanted to test whether *E. coli* that displays rCbpF-1 can activate CEACAM1. For this aim, we used a reporter system that consists of murine thymoma BW cells that were transfected with chimeric proteins composed of the extracellular domain of CEACAM1 fused to a mouse zeta chain. When the CEACAM1 is bound and activated by specific ligands in this system, mouse IL-2 is secreted to the medium. CEACAM1-reporter BW cells were incubated with *E. coli* induced to express *cbpF*-1 or *cbpF*-2. Activation of CEACAM1-expressing BW cells was observed only when incubated with *cbpF*-1-expressing *E. coli* (**Figure 2D**).

## Discussion

CEACAM1 plays an important immunomodulatory role (Gray-Owen and Blumberg, 2006; Kim et al., 2019). Its checkpoint activity is programed to prevent self-harming autoimmune responses. Selection often guides tumors to overexpress CEACAM1 in order to bind and activate CEACAM1 on immune cells and escape anti-tumor activity (Kim et al., 2019). Bacteria have also evolved to express CEACAM1 ligands that assist in attachment and colonization of host tissues (Brewer et al., 2019), but more importantly, impair anti-microbial immunity (Dankner et al., 2017; Gur et al., 2019a; Gur et al., 2019b). As *F. nucleatum* is found in several cancer types (including Colon, esophageal, pancreatic and breast) it is plausible to hypothesize that CEACAM1 activation by tumor-colonized fusobacteria might suppress anti-tumor immunity and affect disease outcome. Therefore, creating tools for analyzing checkpoints activators, that might eventually lead to the generation of novel checkpoint inhibitors is important. Using fusobacterial mutagenesis we recently confirmed that CbpF indeed activates CAECAM1 (Galaski et al., 2021). Here we aimed to generate the first recombinant bacterial immune checkpoint activator. We chose the fusobacterial CbpF, and by optimizing the codon-preference of its gene to that of *E. coli*, and replacing the fusobacterial leader peptide with that of the *E. coli* OmpA, we managed to express a functional full-length trimeric recombinant CbpF-1 on the outer-surface of *E. coli.*

The predicted molecular weight of the rCbpF monomer is 50.5 kDa. SDS-PAGE indicated that rCbpF-1 is in a multimer form (**Figures 1**, **2**). The elution profile of purified rCbpF-1 in gel filtration indicated a high molecular weight, confirming that purified rCbpF-1 is not in a monomeric state (**Supplementary Figure 3**). rCbpF appeared in gels with a higher molecular mass than the predicted molecular weight of the trimer (**Figures 1**, **2**). Such size differences were detectable when rCbpF-1 was subjected to SDS-PAGE gels with different polyacrylamide percentage. In a 6% gel, rCbpF-1 migrated as ~200 kDa (**Figures 1C, D**), while in a 7.5% gel, it migrated as 250 kDa (**Figure 2A**). This observation might point to slight aggregation that occurs during electrophoresis. In native gels, rCbpF-1 migrated at a molecular mass close to the predicted one (**Figure 2B**), suggesting that pre-heating promotes such aggregation due to the exposure of hydrophobic regions (Fink, 1998).

Interestingly, while the rCbpF-2 monomer failed to bind CEACAM1-Ig in the far-western assay, the *E. coli -*bound rCbpF-1 trimer, and the rCbpF1 trimer purified using Ni-NTA from the growth medium, were able to bind CEACAM1-Ig in both denaturing and native far western assays. This might point to a role of the C-terminal autotransporter domain in the proper folding of CbpF and that proper folding of the CEACAM-binding domain of CbpF is required for CEACAM1 binding.

Our results demonstrate the production of the first functional full length CbpF-expressing recombinant *E. coli*, and one of the few full-length recombinant TAAs studied (Grosskinsky et al., 2007; Atack et al., 2020). To the best of our knowledge, it is the only full-length recombinant TAA CEACAM-activating ligand produced (Nordstrom et al., 2004; Tan et al., 2005; Hallstrom et al., 2011; Brewer et al., 2019; Mikula et al., 2019). Recombinant bacterial checkpoint activators might pave the way for future generation of a new class of bacterial-based checkpoints inhibitors.

## Data Availability Statement

The raw data supporting the conclusions of this article will be made available by the authors, without undue reservation.

## Author Contributions

AS and JG designed and carried out experiments, analyzed the data and participated in writing the manuscript. TA-M carried out experiments. JF and RW performed gel-filtration. DS analyzed the data. GB and OM supervised the project and participated in writing the manuscript. All authors contributed to the article and approved the submitted version.

## Funding

This work was supported by the Israel Cancer Research Fund Project grant (GB), the Israel Science Foundation Moked grant and the Israel Ministry of Science and Technology Personalized Medicine grant (GB and OM). AS is supported by the Planning and Budgeting Committee of the Israeli Council for Higher Education. JG is supported by the German Research Foundation (DFG) with a postdoctoral research fellowship.

## Conflict of Interest

The authors declare that the research was conducted in the absence of any commercial or financial relationships that could be construed as a potential conflict of interest.

## Publisher’s Note

All claims expressed in this article are solely those of the authors and do not necessarily represent those of their affiliated organizations, or those of the publisher, the editors and the reviewers. Any product that may be evaluated in this article, or claim that may be made by its manufacturer, is not guaranteed or endorsed by the publisher.

## References

[B1] Atack J. M. Day C. J. Poole J. Brockman K. L. Timms J. R. L. Winter L. E.. (2020). The Nontypeable *Haemophilus influenzae* Major Adhesin Hia Is a Dual-Function Lectin That Binds to Human-Specific Respiratory Tract Sialic Acid Glycan Receptors. mBio 11, e02714–20. 10.1128/mBio.02714-20 33144377 PMC7642680

[B2] Bentancor L. V. Camacho-Peiro A. Bozkurt-Guzel C. Pier G. B. Maira-Litran T. (2012). Identification of Ata, a Multifunctional Trimeric Autotransporter of *Acinetobacter Baumannii* . J. Bacteriol. 194, 3950–3960. 10.1128/JB.06769-11 22609912 PMC3416510

[B3] Brewer M. L. Dymock D. Brady R. L. Singer B. B. Virji M. Hill D. J. (2019). *Fusobacterium* Spp. Target Human CEACAM1 *via* the Trimeric Autotransporter Adhesin CbpF. J. Oral. Microbiol. 11, 1565043. 10.1080/20002297.2018.1565043 30719234 PMC6346709

[B4] Castellarin M. Warren R. L. Freeman J. D. Dreolini L. Krzywinski M. Strauss J.. (2012). *Fusobacterium nucleatum* Infection Is Prevalent in Human Colorectal Carcinoma. Genome Res. 22, 299–306. 10.1101/gr.126516.111 22009989 PMC3266037

[B5] Coppenhagen-Glazer S. Sol A. Abed J. Naor R. Zhang X. Han Y. W.. (2015). Fap2 of *Fusobacterium nucleatum* Is a Galactose-Inhibitable Adhesin Involved in Coaggregation, Cell Adhesion, and Preterm Birth. Infect. Immun. 83, 1104–1113. 10.1128/IAI.02838-14 25561710 PMC4333458

[B6] Dankner M. Gray-Owen S. D. Huang Y. H. Blumberg R. S. Beauchemin N. (2017). CEACAM1 as a Multi-Purpose Target for Cancer Immunotherapy. Oncoimmunology 6, e1328336. 10.1080/2162402X.2017.1328336 28811966 PMC5543821

[B7] Fink A. L. (1998). Protein Aggregation: Folding Aggregates, Inclusion Bodies and Amyloid. Fold. Des. 3, R9–R23. 10.1016/S1359-0278(98)00002-9 9502314

[B8] Galaski A. Shhadeh A. Umaña A. Yoo C. C. Arpinati L. Isaacson B. (2021). *Fusobacterium Nucleatum* CbpF Mediates Inhibition of T Cell Function Through CEACAM1 Activation. Front. Cell Infect. Microbiol. 11, 692544. 10.3389/fcimb.2021.692544 34336716 PMC8319768

[B9] Gray-Owen S. D. Blumberg R. S. (2006). CEACAM1: Contact-Dependent Control of Immunity. Nat. Rev. Immunol. 6, 433–446. 10.1038/nri1864 16724098

[B10] Grosskinsky U. Schutz M. Fritz M. Schmid Y. Lamparter M. C. Szczesny P.. (2007). A Conserved Glycine Residue of Trimeric Autotransporter Domains Plays a Key Role in *Yersinia* Adhesin A Autotransport. J. Bacteriol. 189, 9011–9019. 10.1128/JB.00985-07 17921300 PMC2168626

[B11] Gur C. Ibrahim Y. Isaacson B. Yamin R. Abed J. Gamliel M.. (2015). Binding of the Fap2 Protein of *Fusobacterium nucleatum* to Human Inhibitory Receptor TIGIT Protects Tumors From Immune Cell Attack. Immunity 42, 344–355. 10.1016/j.immuni.2015.01.010 25680274 PMC4361732

[B12] Gur C. Maalouf N. Gerhard M. Singer B. B. Emgard J. Temper V.. (2019a). The *Helicobacter pylori* HopQ Outermembrane Protein Inhibits Immune Cell Activities. Oncoimmunology 8, e1553487. 10.1080/2162402X.2018.1553487 30906650 PMC6422397

[B13] Gur C. Maalouf N. Shhadeh A. Berhani O. Singer B. B. Bachrach G.. (2019b). *Fusobacterium nucleatum* Supresses Anti-Tumor Immunity by Activating CEACAM1. Oncoimmunology 8, e1581531. 10.1080/2162402X.2019.1581531 31069151 PMC6492956

[B14] Hajishengallis G. Lamont R. J. (2016). Dancing With the Stars: How Choreographed Bacterial Interactions Dictate Nososymbiocity and Give Rise to Keystone Pathogens, Accessory Pathogens, and Pathobionts. Trends Microbiol. 24, 477–489. 10.1016/j.tim.2016.02.010 26968354 PMC4874887

[B15] Hallstrom T. Nordstrom T. Tan T. T. Manolov T. Lambris J. D. Isenman D. E.. (2011). Immune Evasion of *Moraxella catarrhalis* Involves Ubiquitous Surface Protein A-Dependent C3d Binding. J. Immunol. 186, 3120–3129. 10.4049/jimmunol.1002621 21270401

[B16] Hammarstrom S. (1999). The Carcinoembryonic Antigen (CEA) Family: Structures, Suggested Functions and Expression in Normal and Malignant Tissues. Semin. Cancer Biol. 9, 67–81. 10.1006/scbi.1998.0119 10202129

[B17] Henderson I. R. Navarro-Garcia F. Desvaux M. Fernandez R. C. Ala’aldeen D. (2004). Type V Protein Secretion Pathway: The Autotransporter Story. Microbiol. Mol. Biol. Rev. 68, 692–744. 10.1128/MMBR.68.4.692-744.2004 15590781 PMC539010

[B18] Hill D. J. Virji M. (2003). A Novel Cell-Binding Mechanism of *Moraxella catarrhalis* Ubiquitous Surface Protein UspA: Specific Targeting of the N-Domain of Carcinoembryonic Antigen-Related Cell Adhesion Molecules by Uspa1. Mol. Microbiol. 48, 117–129. 10.1046/j.1365-2958.2003.03433.x 12657049

[B19] Hill D. J. Whittles C. Virji M. (2012). A Novel Group of *Moraxella Catarrhalis* UspA Proteins Mediates Cellular Adhesion *via* CEACAMs and Vitronectin. PloS One 7, e45452. 10.1371/journal.pone.0045452 23049802 PMC3458076

[B20] Javaheri A. Kruse T. Moonens K. Mejias-Luque R. Debraekeleer A. Asche C. I.. (2016). *Helicobacter pylori* Adhesin HopQ Engages in a Virulence-Enhancing Interaction With Human CEACAMs. Nat. Microbiol. 2, 16189. 10.1038/nmicrobiol.2016.189 27748768

[B21] Kapatral V. Anderson I. Ivanova N. Reznik G. Los T. Lykidis A.. (2002). Genome Sequence and Analysis of the Oral Bacterium *Fusobacterium nucleatum* Strain ATCC 25586. J. Bacteriol. 184, 2005–2018. 10.1128/JB.184.7.2005-2018.2002 11889109 PMC134920

[B22] Kim W. M. Huang Y. H. Gandhi A. Blumberg R. S. (2019). CEACAM1 Structure and Function in Immunity and Its Therapeutic Implications. Semin. Immunol. 42, 101296. 10.1016/j.smim.2019.101296 31604530 PMC6814268

[B23] Koniger V. Holsten L. Harrison U. Busch B. Loell E. Zhao Q.. (2016). *Helicobacter pylori* Exploits Human CEACAMs *via* HopQ for Adherence and Translocation of CagA. Nat. Microbiol. 2, 16188. 10.1038/nmicrobiol.2016.188 27748756

[B24] Kostic A. D. Chun E. Robertson L. Glickman J. N. Gallini C. A. Michaud M.. (2013). *Fusobacterium nucleatum* Potentiates Intestinal Tumorigenesis and Modulates the Tumor-Immune Microenvironment. Cell Host Microbe 14, 207–215. 10.1016/j.chom.2013.07.007 23954159 PMC3772512

[B25] Kostic A. D. Gevers D. Pedamallu C. S. Michaud M. Duke F. Earl A. M.. (2012). Genomic Analysis Identifies Association of *Fusobacterium* With Colorectal Carcinoma. Genome Res. 22, 292–298. 10.1101/gr.126573.111 22009990 PMC3266036

[B26] Lamont R. J. Koo H. Hajishengallis G. (2018). The Oral Microbiota: Dynamic Communities and Host Interactions. Nat. Rev. Microbiol. 16, 745–759. 10.1038/s41579-018-0089-x 30301974 PMC6278837

[B27] Leo J. C. Elovaara H. Brodsky B. Skurnik M. Goldman A. (2008). The *Yersinia* Adhesin YadA Binds to a Collagenous Triple-Helical Conformation But Without Sequence Specificity. Protein Eng. Des. Sel. 21, 475–484. 10.1093/protein/gzn025 18467342 PMC3254170

[B28] Leo J. C. Goldman A. (2009). The Immunoglobulin-Binding Eib Proteins From *Escherichia coli* Are Receptors for IgG Fc. Mol. Immunol. 46, 1860–1866. 10.1016/j.molimm.2009.02.024 19303642

[B29] Leo J. C. Grin I. Linke D. (2012). Type V Secretion: Mechanism(s) of Autotransport Through the Bacterial Outer Membrane. Philos. Trans. R. Soc. Lond. B. Biol. Sci. 367, 1088–1101. 10.1098/rstb.2011.0208 22411980 PMC3297439

[B30] Leo J. C. Lyskowski A. Hattula K. Hartmann M. D. Schwarz H. Butcher S. J.. (2011). The Structure of *E. Coli* IgG-Binding Protein D Suggests a General Model for Bending and Binding in Trimeric Autotransporter Adhesins. Structure 19, 1021–1030. 10.1016/j.str.2011.03.021 21742268

[B31] Markel G. Wolf D. Hanna J. Gazit R. Goldman-Wohl D. Lavy Y.. (2002). Pivotal Role of CEACAM1 Protein in the Inhibition of Activated Decidual Lymphocyte Functions. J. Clin. Invest. 110, 943–953. 10.1172/JCI0215643 12370272 PMC151149

[B32] Mikula K. M. Kolodziejczyk R. Goldman A. (2019). Structure of the UspA1 Protein Fragment From *Moraxella catarrhalis* Responsible for C3d Binding. J. Struct. Biol. 208, 77–85. 10.1016/j.jsb.2019.08.002 31400508 PMC6839023

[B33] Mima K. Nishihara R. Qian Z. R. Cao Y. Sukawa Y. Nowak J. A.. (2016). *Fusobacterium nucleatum* in Colorectal Carcinoma Tissue and Patient Prognosis. Gut 65, 1973–1980. 10.1136/gutjnl-2015-310101 26311717 PMC4769120

[B34] Mima K. Sukawa Y. Nishihara R. Qian Z. R. Yamauchi M. Inamura K.. (2015). *Fusobacterium nucleatum* and T Cells in Colorectal Carcinoma. JAMA Oncol. 1 (5), 653–661. 10.1001/jamaoncol.2015.1377 26181352 PMC4537376

[B35] Mitsuhashi K. Nosho K. Sukawa Y. Matsunaga Y. Ito M. Kurihara H.. (2015). Association of *Fusobacterium* Species in Pancreatic Cancer Tissues With Molecular Features and Prognosis. Oncotarget 6, 7209–7220. 10.18632/oncotarget.3109 25797243 PMC4466679

[B36] Nordstrom T. Blom A. M. Forsgren A. Riesbeck K. (2004). The Emerging Pathogen *Moraxella catarrhalis* Interacts With Complement Inhibitor C4b Binding Protein Through Ubiquitous Surface Proteins A1 and A2. J. Immunol. 173, 4598–4606. 10.4049/jimmunol.173.7.4598 15383594

[B37] Nozawa A. Oshima H. Togawa N. Nozaki T. Murakami S. (2020). Development of Oral Care Chip, a Novel Device for Quantitative Detection of the Oral Microbiota Associated With Periodontal Disease. PloS One 15, e0229485. 10.1371/journal.pone.0229485 32109938 PMC7048280

[B38] Obrink B. (1997). CEA Adhesion Molecules: Multifunctional Proteins With Signal-Regulatory Properties. Curr. Opin. Cell Biol. 9, 616–626. 10.1016/S0955-0674(97)80114-7 9330864 PMC7135799

[B39] Parhi L. Alon-Maimon T. Sol A. Nejman D. Shhadeh A. Fainsod-Levi T.. (2020). Breast Cancer Colonization by *Fusobacterium nucleatum* Accelerates Tumor Growth and Metastatic Progression. Nat. Commun. 11, 3259. 10.1038/s41467-020-16967-2 32591509 PMC7320135

[B40] Prall F. Nollau P. Neumaier M. Haubeck H. D. Drzeniek Z. Helmchen U.. (1996). CD66a (BGP), an Adhesion Molecule of the Carcinoembryonic Antigen Family, Is Expressed in Epithelium, Endothelium, and Myeloid Cells in a Wide Range of Normal Human Tissues. J. Histochem. Cytochem. 44, 35–41. 10.1177/44.1.8543780 8543780

[B41] Riess T. Andersson S. G. Lupas A. Schaller M. Schafer A. Kyme P.. (2004). *Bartonella* Adhesin a Mediates a Proangiogenic Host Cell Response. J. Exp. Med. 200, 1267–1278. 10.1084/jem.20040500 15534369 PMC2211922

[B42] Socransky S. S. Haffajee A. D. Cugini M. A. Smith C. Kent R. L. Jr. (1998). Microbial Complexes in Subgingival Plaque. J. Clin. Periodontol. 25, 134–144. 10.1111/j.1600-051X.1998.tb02419.x 9495612

[B43] Szczesny P. Linke D. Ursinus A. Bar K. Schwarz H. Riess T. M.. (2008). Structure of the Head of the *Bartonella* Adhesin BadA. PloS Pathog. 4, e1000119. 10.1371/journal.ppat.1000119 18688279 PMC2483945

[B44] Tan T. T. Nordstrom T. Forsgren A. Riesbeck K. (2005). The Respiratory Pathogen *Moraxella catarrhalis* Adheres to Epithelial Cells by Interacting With Fibronectin Through Ubiquitous Surface Proteins A1 and A2. J. Infect. Dis. 192, 1029–1038. 10.1086/432759 16107956

[B45] Tegtmeyer N. Harrer A. Schmitt V. Singer B. B. Backert S. (2019). Expression of CEACAM1 or CEACAM5 in AZ-521 Cells Restores the Type IV Secretion Deficiency for Translocation of CagA by *Helicobacter Pylori* . Cell Microbiol. 21, e12965. 10.1111/cmi.12965 30321907

[B46] Wang N. Feng Y. Wang Q. Liu S. Xiang L. Sun M.. (2014). Neutrophils Infiltration in the Tongue Squamous Cell Carcinoma and Its Correlation With CEACAM1 Expression on Tumor Cells. PloS One 9, e89991. 10.1371/journal.pone.0089991 24587171 PMC3937421

[B47] Yamamura K. Baba Y. Nakagawa S. Mima K. Miyake K. Nakamura K.. (2016). Human Microbiome *Fusobacterium nucleatum* in Esophageal Cancer Tissue Is Associated With Prognosis. Clin. Cancer Res. 22, 5574–5581. 10.1158/1078-0432.CCR-16-1786 27769987

[B48] Yamaoka Y. Suehiro Y. Hashimoto S. Hoshida T. Fujimoto M. Watanabe M.. (2018). *Fusobacterium nucleatum* as a Prognostic Marker of Colorectal Cancer in a Japanese Population. J. Gastroenterol. 53, 517–524. 10.1007/s00535-017-1382-6 28823057

[B49] Yamin R. Lecker L. S. M. Weisblum Y. Vitenshtein A. Le-Trilling V. T. K. Wolf D. G.. (2016). HCMV Vcxcl1 Binds Several Chemokine Receptors and Preferentially Attracts Neutrophils Over NK Cells by Interacting With CXCR2. Cell Rep. 15, 1542–1553. 10.1016/j.celrep.2016.04.042 27160907

[B50] Zhang Y. Cai P. Li L. Shi L. Chang P. Liang T.. (2017). Co-Expression of TIM-3 and CEACAM1 Promotes T Cell Exhaustion in Colorectal Cancer Patients. Int. Immunopharmacol. 43, 210–218. 10.1016/j.intimp.2016.12.024 28038383

