# Supplementary material for: CEACAM1 Activation by CbpF-Expressing E. coli
Source: Front Cell Infect Microbiol. 2021 Jul 29;11:699015. doi: 10.3389/fcimb.2021.699015 (PMC8358318; doi:10.3389/fcimb.2021.699015)
Supplement: Supplementary file 1 [file DataSheet_1.docx]

**Supplementary Material**

**
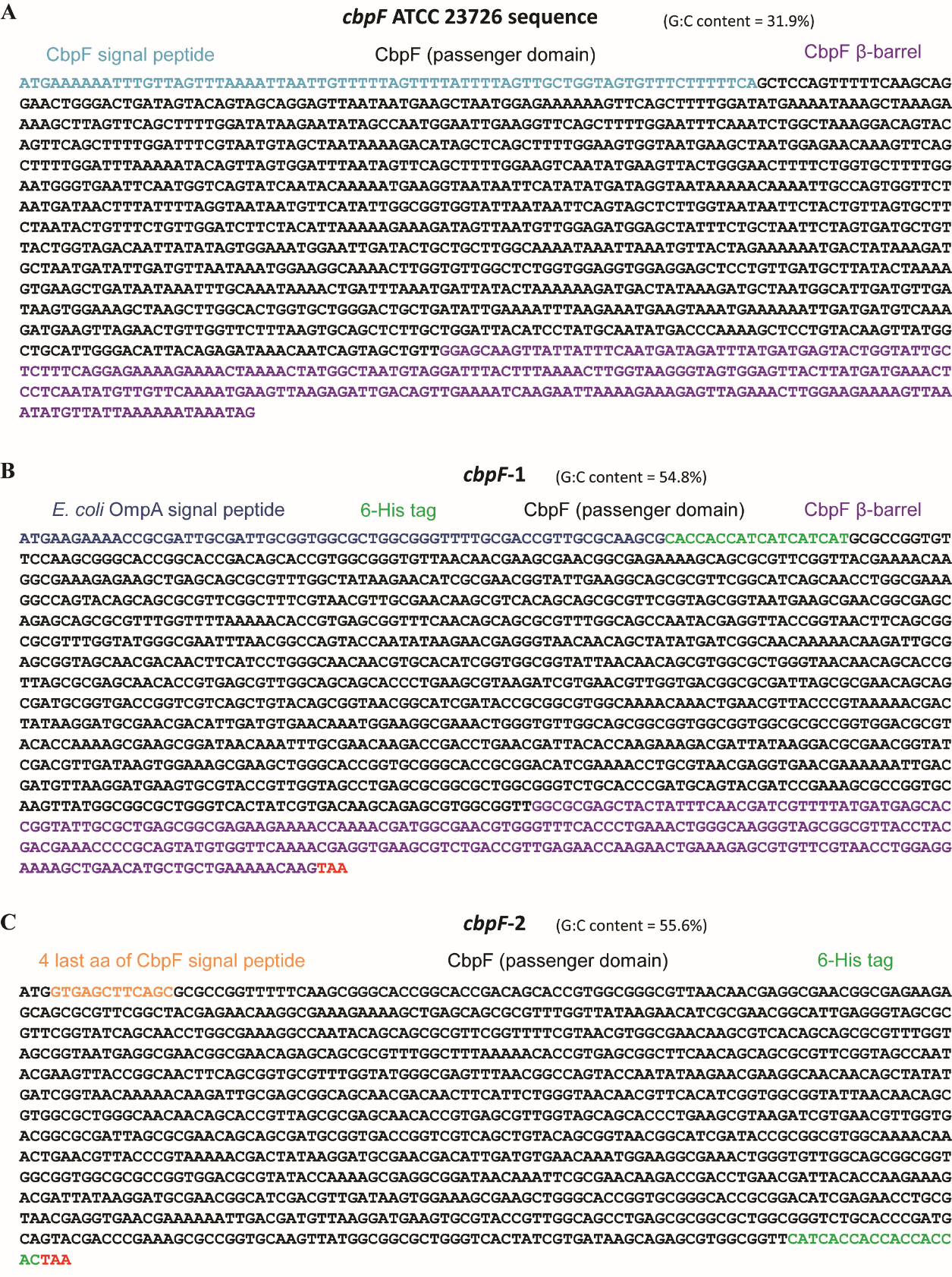
**

**Supplementary Figure 1. *cbpF* gene optimization for expression in *E. coli*.** (**A**) The *F. nucleatum* ATCC 23726 gene-encoding CbpF (*cbpF,* GenBank: AVQ22222.1). (**B**) For constructing rCbpF-1, CbpF’s signal peptide (amino acids 1-24) was replaced with the one of *E. coli* OmpA, followed by 6 histidines, and by the *cbpF* gene coding for amino acids 25-492 using *E. coli* codon preference. (**C**) For constructing rCbpF-2, the last four amino acids of CbpF’s signal peptide (21-24) were kept, followed by the *cbp*F gene coding amino acids 25-408 (using *E. coli* codon preference) ending with a C-terminal His-tag. rCbpF-2 lacks a signal peptide and the CbpF β-barrel autotransporter.


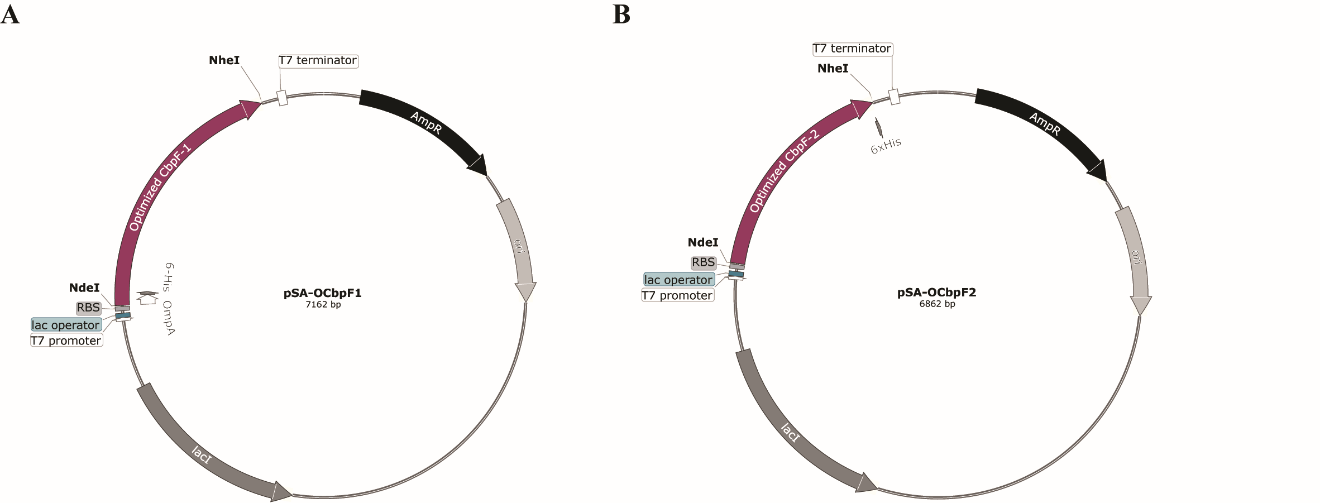


**Supplementary Figure 2.** Schematic representation of the optimized *cbpF*-1 (A) and *cbpF*-2 (B) expressing plasmids pSA-OCbpF1 and pSA-OCbpF2 respectively.


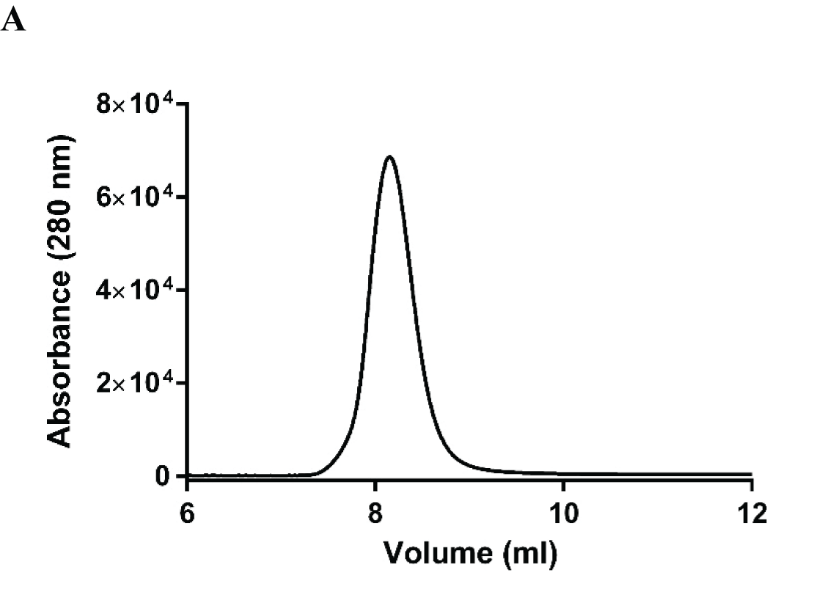


**Supplementary Figure 3. Purified rCbpF-1 -size exclusion chromatography (SEC).** 40 µl of purified rCbpF-1 (850 µg/ml) where injected to an analytical SEC column (Superdex 200 10/300 GL; GE Healthcare Life Sciences) equilibrated with a buffer containing 20 mM Tris (pH 7.5), 150 mM NaCl and 10% glycerol. Absorbance plotted against elution volume.
